# Infection with Panton-Valentine Leukocidin–Positive Methicillin-Resistant *Staphylococcus aureus* t034

**DOI:** 10.3201/eid1408.071427

**Published:** 2008-08

**Authors:** Christina Welinder-Olsson, Kerstin Florén-Johansson, Leif Larsson, Sven Öberg, Lisbeth Karlsson, Christina Åhrén

**Affiliations:** * Sahlgrenska University Hospital, Göteborg, Sweden; † Uddevalla Hospital, Uddevalla, Sweden; ‡ Borås Hospital, Borås, Sweden

**Keywords:** MRSA, t034, PVL, ST398, dispatch

## Abstract

Panton-Valentine leukocidin (PVL)–positive methicillin-resistant *Staphylococcus aureus* (MRSA), sequence type 398 is believed to be of animal origin. We report 2 cases of infection due to PVL–positive MRSA, *spa* type t034, in patients in Sweden who had had no animal contact.

The problem of methicillin-resistant *Staphylococcus aureus* (MRSA) is increasing worldwide. MRSA is no longer restricted to hospital settings but is found in homes, places of work, and kindergartens. Cases of animal MRSA infection and carriage are increasingly reported and cause substantial occupational health problems for farmers and veterinary staff (*1*–*3*).

MRSA carriage by domestic animals has been recently reported (*3*–*5*). In the Netherlands in 2005, MRSA of the lineage sequence type (ST) 398, *spa* type t108, was detected and found to be transmittable between animals and humans, e.g., pigs and pig farmers, as well as between humans (*2*). This zoonotic potential has warranted alertness to ask about animal contact when screening patients for MRSA.

Additional *spa* types of the clonal lineage ST398 have been identified among humans and animals. Witte et al. reported on *spa* types t011, t034, and t1197, isolated from colonized and infected humans and companion animals (e.g., dog, pig, horse) in Germany and Austria (*5*). Recently, farmers and veterinarians from 7 countries were found to carry MRSA strains of these *spa* types (*1*). Even more alarming are recent reports of these strains causing serious infection in humans (*4*–*6*). A common trait reported for the strains in the clonal lineage ST398 is the indigestibility of their whole cellular DNA when subjected to *Sma*I-macrorestriction analysis and their consequent inability to be typed by pulsed-field gel electrophoresis (PFGE) (*5*,*7*).

## The Cases

We report 2 patients infected with Panton-Valentine leukocidin (PVL)–positive MRSA t034. Each patient had a medical history typical of that reported for community-acquired MRSA of other lineages, which in most cases are PVL positive (*8*).

The first patient, a previously healthy 36-year-old male physiotherapist, sought medical care in March 2006 for a small abscess in his axilla. Culture of the abscess grew MRSA. Presence of m*ecA* gene was confirmed by PCR (*9*). During the next 2 months, furunculous developed twice, caused by the same strain. His youngest child, adopted from China, had been found to be MRSA positive (throat, perineum, and a small wound) a month earlier during routine screening for adopted children. During subsequent screening of the family, the older sister, adopted from South Korea, was also found positive (throat). Both parents were negative for MRSA at that time, which suggests that the father was newly infected when his abscess developed and that he had not acquired the strain abroad. Also, *spa* typing indicated that the children carried different strains from that of the father and from each other (t286, t1434) (*10*). Subsequent screening of family members for MRSA on several occasions found only the father to be repeatedly positive.

The second patient, a 43-year-old male clerk, also previously healthy, sought medical attention during the summer of 2007 for a MRSA-infected elbow wound. Follow-up examination determined that he carried MRSA also in the perineum and in a chronic external otitis eczema. He was later hospitalized for a larger abscess that required surgical drainage. His family members reported no symptoms and were thus not screened for MRSA.

The patients lived in geographically distinct areas in the western part of Sweden and had no connection to each other. No animal contact (e.g., pets, farming) was reported by the 2 patients, their family members, or other close contacts.

Both patient strains carried PVL, confirmed by identification of the *lukS-lukF* genes (*11*), and were resistant to digestion with restriction endonuclease *Sma*I when typing by PFGE was attempted. Both belonged to the t034 *spa* type. They produced β-hemolysin according to phenotypic detection methods that used rabbit blood agar with hot–cold analysis, which further indicated their animal origin (*12*). Their drug-susceptibility profiles differed; 1 was resistant to doxycycline and the other was resistant to ciprofloxacin, erythromycin, and clindamycin.

## Conclusions

These strains carry PVL, a toxin partly responsible for the increased virulence of several of the MRSA clones in the community (*8*). Despite several recent publications concerning ST398 MRSA, few have reported PVL in this lineage, which is believed to be of animal origin (*2*,*4*,*6*,*13*). Most previous reports have described asymptomatic carriage in persons exposed to occupational hazards (e.g., veterinary personal and pig farmers) (*1*,*2*,*7*). However, severe clinical infections have been described (*4*–*6*). In our patients these strains caused repeated infections that needed medical attention, even hospitalization. Since neither patient had even a remote connection to animals and we found no common source of infection, these strains may already be more common in our region than we had thought. These case reports suggest that strains of this lineage may impose a threat in the community, even to patients with no obvious animal contact.

## References

[1] Wulf MW, Sorum M, van Nes A, Skov R, Melchers WJ, Klaassen CH, Prevalence of methicillin-resistant *Staphylococcus aureus* among veterinarians: an international study. Clin Microbiol Infect. 2008;14:29–34. 10.1111/j.1469-0691.2007.01873.x 17986212

[2] Voss A, Loeffen F, Bakker J, Klaassen C, Wulf M. Methicillin-resistant *Staphylococcus aureus* in pig farming. Emerg Infect Dis. 2005;11:1965–6. 16485492 10.3201/eid1112.050428 PMC3367632

[3] Weese JS, Archambault M, Willey BM, Hearn P, Kreiswirth BN, Said-Salim B, Methicillin-resistant *Staphylococcus aureu*s in horses and horse personnel, 2000–2002. Emerg Infect Dis. 2005;11:430–5. 15757559 10.3201/eid1103.040481 PMC3298236

[4] van Belkum A, Melles DC, Peeters JK, van Leeuwen WB, van Duijkeren E, Huijsdens XW, Methicillin-resistant and -susceptible *Staphylococcus aureus* sequence type 398 in pigs and humans. Emerg Infect Dis. 2008;14:479–83. 10.3201/eid1403.0760 18325267 PMC2570802

[5] Witte W, Strommenger B, Stanek C, Cuny C. Methicillin-resistant *Staphylococcus aureus* ST398 in humans and animals, Central Europe. Emerg Infect Dis. 2007;13:255–8. 17479888 10.3201/eid1302.060924 PMC2725865

[6] Yu F, Chen Z, Liu C, Zhang X, Lin X, Chi S, Prevalence of *Staphylococcus aureus* carrying Panton-Valentine leukocidin genes among isolates from hospitalised patients in China. Clin Microbiol Infect. 2008;14:381–4. 10.1111/j.1469-0691.2007.01927.x 18190580

[7] Bens CC, Voss A, Klaassen CH. Presence of a novel DNA methylation enzyme in methicillin-resistant *Staphylococcus aureus* isolates associated with pig farming leads to uninterpretable results in standard pulsed-field gel electrophoresis analysis. J Clin Microbiol. 2006;44:1875–6. 10.1128/JCM.44.5.1875-1876.2006 16672428 PMC1479204

[8] Boyle-Vavra S, Daum RS. Community-acquired methicillin-resistant *Staphylococcus aureus:* the role of Panton-Valentine leukocidin. Lab Invest. 2007;87:3–9. 10.1038/labinvest.3700501 17146447

[9] Geha DJ, Uhl JR, Gustaferro CA, Persing DH. Multiplex PCR for identification of methicillin-resistant staphylococci in the clinical laboratory. J Clin Microbiol. 1994;32:1768–72. 7929772 10.1128/jcm.32.7.1768-1772.1994 PMC263789

[10] Harmsen D, Claus H, Witte W, Rothganger J, Turnwald D, Vogel U. Typing of methicillin-resistant *Staphylococcus aureus* in a university hospital setting by using novel software for *spa* repeat determination and database management. J Clin Microbiol. 2003;41:5442–8. 10.1128/JCM.41.12.5442-5448.2003 14662923 PMC309029

[11] Lina G, Piemont Y, Godail-Gamot F, Bes M, Peter MO, Gauduchon V, Involvement of Panton-Valentine leukocidin-producing *Staphylococcus aureus* in primary skin infections and pneumonia. Clin Infect Dis. 1999;29:1128–32. 10.1086/313461 10524952

[12] Monecke S, Kuhnert P, Hotzel H, Slickers P, Ehricht R. Microarray based study on virulence-associated genes and resistance determinants of *Staphylococcus aureus* isolates from cattle. Vet Microbiol. 2007;125:128–40. 10.1016/j.vetmic.2007.05.016 17614219

[13] Wulf M, van Nes A, Eikelenboom-Boskamp A, de Vries J, Melchers W, Klaassen C, Methicillin-resistant *Staphylococcus aureus* in veterinary doctors and students, the Netherlands. Emerg Infect Dis. 2006;12:1939–41. 17326948 10.3201/eid1212.060355 PMC3291345

